# *verA* Gene is Involved in the Step to Make the Xanthone Structure of Demethylsterigmatocystin in Aflatoxin Biosynthesis

**DOI:** 10.3390/ijms21176389

**Published:** 2020-09-02

**Authors:** Hongmei Zeng, Jingjing Cai, Hidemi Hatabayashi, Hiroyuki Nakagawa, Hiromitsu Nakajima, Kimiko Yabe

**Affiliations:** 1 Food Research Institute, National Agriculture and Food Research Organization (NARO), 2-1-12 Kannon-dai, Tsukuba-shi 305-8642, Ibaraki, Japan; zenghongmei@caas.cn (H.Z.); caijingjing@caas.cn (J.C.); hata-shhkyt0109@docomo.ne.jp (H.H.); hironkgw@affrc.go.jp (H.N.); 2 Institute of Plant Protection, Chinese Academy of Agricultural Sciences, Beijing 100193, China; 3 Biotechnology Research Institute, Chinese Academy of Agricultural Sciences, Beijing 100081, China; 4 Faculty of Agriculture, Tottori University, Koyama, Tottori 680-8553, Japan; nakajima@tottori-u.ac.jp; 5 Department of Applied Chemistry and Food Science, Faculty of Environmental and Information Sciences, Fukui University of Technology, 3-6-1 Gakuen, Fukui-shi, Fukui 910-8505, Japan

**Keywords:** aflatoxin biosynthesis, enzyme gene, *dmtA* (*aflO*), *hypA* (*aflY*), *ordB* (*aflX*), HAMA intermediate, *stcP*, *verA* (*aflN*), *ver-1* (*aflM*)

## Abstract

In the biosynthesis of aflatoxin, *verA*, *ver-1*, *ordB*, and *hypA* genes of the aflatoxin gene cluster are involved in the pathway from versicolorin A (VA) to demethylsterigmatocystin (DMST). We herein isolated each disruptant of these four genes to determine their functions in more detail. Disruptants of *ver-1*, *ordB*, and *hypA* genes commonly accumulated VA in their mycelia. In contrast, the *verA* gene disruptant accumulated a novel yellow fluorescent substance (which we named HAMA) in the mycelia as well as culture medium. Feeding HAMA to the other disruptants commonly caused the production of aflatoxins B_1_ (AFB_1_) and G_1_ (AFG_1_). These results indicate that HAMA pigment is a novel aflatoxin precursor which is involved at a certain step after those of *ver-1*, *ordB*, and *hypA* genes between VA and DMST. HAMA was found to be an unstable substance to easily convert to DMST and sterigmatin. A liquid chromatography-mass spectrometry (LC-MS) analysis showed that the molecular mass of HAMA was 374, and HAMA gave two close major peaks in the LC chromatogram in some LC conditions. We suggest that these peaks correspond to the two conformers of HAMA; one of them would be selectively bound on the substrate binding site of VerA enzyme and then converted to DMST. VerA enzyme may work as a key enzyme in the creation of the xanthone structure of DMST from HAMA.

## 1. Introduction

Aflatoxins (AFs) are highly toxic, carcinogenic, teratogenic, and mutagenic secondary metabolites produced primarily by certain strains of *Aspergillus flavus* and *Aspergillus parasiticus* [1]. Other strains (*A. nomius*, *A. pseudotamarii*, *A. bombycis* and more) have also been reported to produce AFs, and aflatoxigenic fungi were shown to be widely distributed mainly in tropical and subtropical regions [2,3,4,5,6]. The AF contamination of crops such as corn, cotton, and nuts has serious effects on the health of animals and humans, and it also causes economic problems due to crop losses [7,8,9].

Aflatoxigenic fungi can potentially produce eight types of AF: the four major AFs, i.e., aflatoxins B_1_ (AFB_1_), B_2_ (AFB_2_), G_1_ (AFG_1_), and G_2_ (AFG_2_) and the four minor AFs, aflatoxins M_1_ (AFM_1_), M_2_ (AFM_2_), GM_1_ (AFGM_1_), and GM_2_ (AFGM_2_) [10,11]. The former AFs AFB_1_, AFB_2_, AFG_1_ and AFG_2_ are major AFs because of their high quantities. Among them, AFB_1_ is the most potent carcinogenic and toxic substance, and it is classified as group 1 (a human carcinogen) by the International Agency for Research on Cancer [12]. The contamination of crops and food products with the major AFs is a very serious problem in food safety [9].

**Figure 1:**
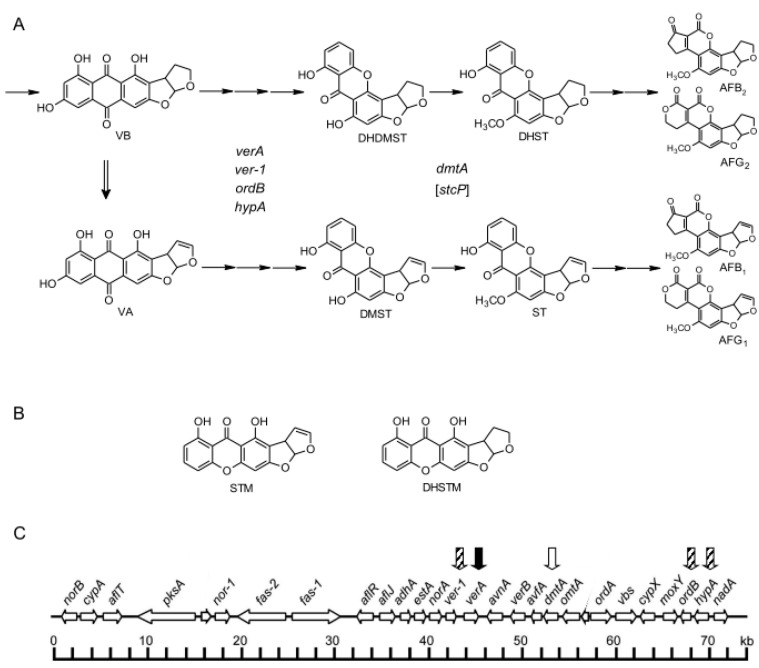
Outline of the biosynthetic pathway of aflatoxins (AFs) and the AF gene cluster. (**A**) The formations of 1-group AFs (AFB_1_ and AFG_1_) from VA and 2-group AFs (AFB_2_ and AFG_2_) from VB. In the biosynthesis of AF, there are pathways for the formation of 1-group AFs (AFB_1_ and AFG_1_) from VA and 2-group AFs (AFB_2_ and AFG_2_) from VB. VB is converted to VA, and VA and VB respectively serve as precursors of 1-group and 2-group AFs. *ver-1* (*aflM*), *ordB* (*aflX*), *hypA* (*aflY*), and *verA* (*aflN*) genes are suspected to be involved in the step from VA to DMST as well as the step from VB to DHDMST. *dmtA* (*aflO*) gene, which is a homolog of *A. nidulans stcP* gene, is involved in the conversion from DMST to ST as well as the conversion from DHDMST to DHST. (**B**) The structures of STM and DHSTM. STM is the stereoisomer of DMST, and DHSTM is the stereoisomer of DHDMDT. (**C**) The AF gene cluster in the genome of aflatoxigenic fungus. Enzyme genes and regulatory gene(s) are schematically shown in an approx. 70-kb DNA region. *verA* gene (closed arrow) and each of *ver-1*, *ordB* and *hypA* genes (striped arrows), and *dmtA* gene (open arrow) are indicated. DHDMST: dihydrodemethylsterigmatocystin, DHST: dihydrosterigmatocystin, DHSTM: dihydrosterigmatocystin, DMST: demethylsterigmatocystin, ST: sterigmatocystin, STM: sterigmatin, VA: versicolorin A, VB: versicolorin B.

All AFs are produced from acetyl-CoA through complicated branching pathways involving more than 25 enzymatic reactions [13,14,15,16,17]. The following outline of the pathway has been proposed: acetyl-CoA→polyketide→norsolorinic acid (NA)→averantin (AVN)→averufin (AVR)→hydroxyversicolorone (HVN)→versiconal hemiacetal acetate (VHA)→versiconal (VHOH)→versicolorin B (VB)→versicolorin A (VA)→demethylsterigmatocystin (DMST)→ST→*O*-methysterigmatocystin (OMST)→AFB_1_ and AFG_1_. In the pathway, the formation of VA from VB by a desaturase is a branching step for the formation of all 1-group AFs such as AFB_1_ and AFG_1_ and that of all 2-group AFs such as AFB_2_ and AFG_2_ (Figure 1A) [11,18]. CypA and OrdA enzymes catalyze the branching step for the formation of a G-group AF from the B-group AF formation pathway [19,20,21]. OrdA enzyme also catalyze the branching steps for the formation of M-group AFs (AFM_1_ and AFM_2_) from the B-group AF formation pathway and for the formation of GM-group AFs (AFGM_1_ and AFGM_2_) from the G-group AF formation pathway [10].

*A. nidulans, A. versicolor* and various fungal species are known to produce sterigmatocystin (ST) as a final product [21,22,23,24,25,26]. ST is one of the latter intermediates in the biosynthesis of AF. In fact, the enzymes, the intermediates, and the reactions for the biosynthesis of ST in *A. nidulans* are the same as those for AF biosynthesis [10,16,25].

At least 25 genes coding for the enzymes and regulatory factor(s) needed for AF biosynthesis are clustered in a 70-kb DNA region (Figure 1C) [10,16,17]. Although functions of most of these genes have been clarified, some remain to be identified. The pathway from VA to DMST is one of these undetermined steps. Enzymes encoded by *hypA* (*aflY*) [27], *ordB* (*aflX*) [28], and *ver-1* (*aflM*) [29] are known to be involved in the pathway, as the deletion of each of them caused an accumulation of VA with a remarkable decrease or loss of AF production (Figure 1A). The *verA* (*aflN*) gene of *A. parasiticus* is suspected to be involved in the conversion of VA to DMST, as the deletion of *stcS* gene of *A. nidulans* caused an accumulation of VA in its mycelia with a decrease in the production of ST [30], and the *A. nidulans stcS* gene was suggested to be a homolog of *verA* gene based on the relatively high similarity (75%) of their amino acid sequences [16].

Hamasaki et al. [31] first isolated sterigmatin (STM) as a metabolite of *A. versicolor* (Vuillemin) Tiraboschi, and they suggested that STM is an isomer of DMST because STM has a linear [3,2-b]fusion structure of the xanthone and dihydrobisfuran moieties (Figure 1B), whereas DMST has a bending [2,3-c]fusion structure of the same moieties [32]. Fukuyama et al. also suggested that STM is produced from VA, and that a putative precursor might work to make either DMST or STM, which depends on the manner of ether linkage formation to build a xanthone ring [33]. Austocystins A–I, which are secondary metabolites produced by *Aspergillus ustus*, have linear structures that are similar to that of STM, and they have been proposed to be derived from VA [34].

In the biosynthesis of AFs, the xanthone formation step corresponds to the production of carcinogenicity; ST derivatives and AFs are known to have carcinogenicity and acute toxicity [22,35]. In contrast, anthraquinone derivatives such as VA and VB have low or not-yet-identified toxicity [36]. To clarify the important step of the appearance of toxicities, we herein investigated the pathway from VA to DMST in detail. In this work, we isolated each disruptant of *ver-1*, *hypA, ordB*, and *verA* genes (Figure 1C) and then characterized the accumulating substances in the culture medium or mycelia. The results demonstrated that: (1) *verA* disruptant accumulated mostly a novel intermediate, which we named HAMA after the late Professor Takashi Hamasaki, and (2) the *verA* gene is involved in the pathway from HAMA to DMST through a regiospecific xanthone ring formation. We also investigated accumulating substances in the deletion mutant of *A. nidulan*s *stcP* gene [37], which is a homologue of the *dmtA* (*aflO*) gene of *A. parasiticus* [38]. These genes are involved in the reaction from DMST to ST. We evaluated these substances to determine whether HAMA is produced before the formation of DMST in AF biosynthesis as well as ST biosynthesis. We propose possible reaction steps in the pathway from VA to DMST.

## 2. Results

### 2.1. Preparation of verA Disruptant

**Figure 2:**
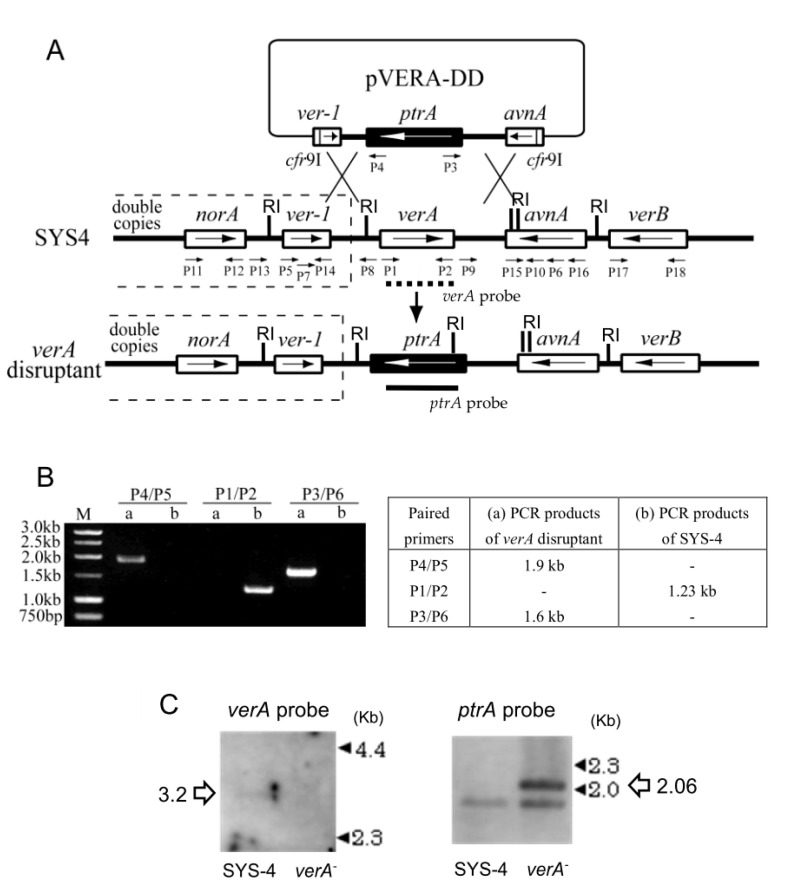
Disruption of *verA* gene. (**A**) The strategy for the disruption of the *verA* gene. The double-crossover recombination events resulted in the replacement of the *verA* gene with the selectable marker *ptrA* gene. Long arrows: gene direction, short arrows: position of primers used for the confirmation of gene disruption. (**B**) A PCR analysis using different combinations of primers was done to confirm whether the *verA* gene was deleted in the *verA* disruptant. The PCR products obtained with the primer pairs (as shown in the table) were used to confirm the *verA* disruption. M, 1-kb molecular marker. (**C**) Southern analysis of the *verA* disruptant. Genomic DNA of strain SYS4 (wild stain) or the *verA* disruptant were digested with *Eco*RI and analyzed by Southern hybridization using the *verA* probe (left) or *ptrA* probe (right). The positions of the size markers are indicated by closed triangles. The sizes of the resulting fragments are indicated by open arrows.

*verA* gene of *A. parasiticus* SYS4 was disrupted via the double-crossover strategy by using the linear DNA fragments of pVERA-DD, which was constructed with the flanking sequences of *verA* and disruption vector (Figure 2A). The *verA* disruptant was then isolated through the transformation of SYS4 with the linear DNA fragments of pVERA-DD. We obtained six mutants, three of which were then confirmed to be the right disruptants by PCRs using primer pairs P4/P5, P1/P2, and P3/P6 (Figure 2B) (Table 1) and a Southern analysis (Figure 2C). In the latter analysis, the *verA* gene coding region was presented on an approx. 3.20 kb *Eco*RI fragment of the wildtype SYS4 genome, whereas *verA* disruptant DNA gave no hybridization band (Figure 2C, Left). When *ptrA* gene was used as a probe, beside the band that was suspected to correspond to the putative homologous *nmt-1* gene in the *A. parasiticus* genome [39], specific *ptrA* hybridization signals of 2.06 kb for *Eco*RI digestion were detected only in the genomic DNA of *verA* disruptant (Figure 2C, Right). These results indicate that the *verA* gene was replaced with the *ptrA*-selectable marker in the genomes of the mutants.

Colonies of *verA* disruptant did not show any clear difference in phenotype such as mycelia color or size from those of the wildtype. In contrast, the colonies on the GY agar plate showed white images on UV photos, indicating that the resulting mutants had lost or decreased AF productivity. *verA* gene was thus suggested to be involved in the biosynthesis of AFs.

### 2.2. Characterizations of the Substances Accumulating in the verA Disruptant

**Figure 3:**
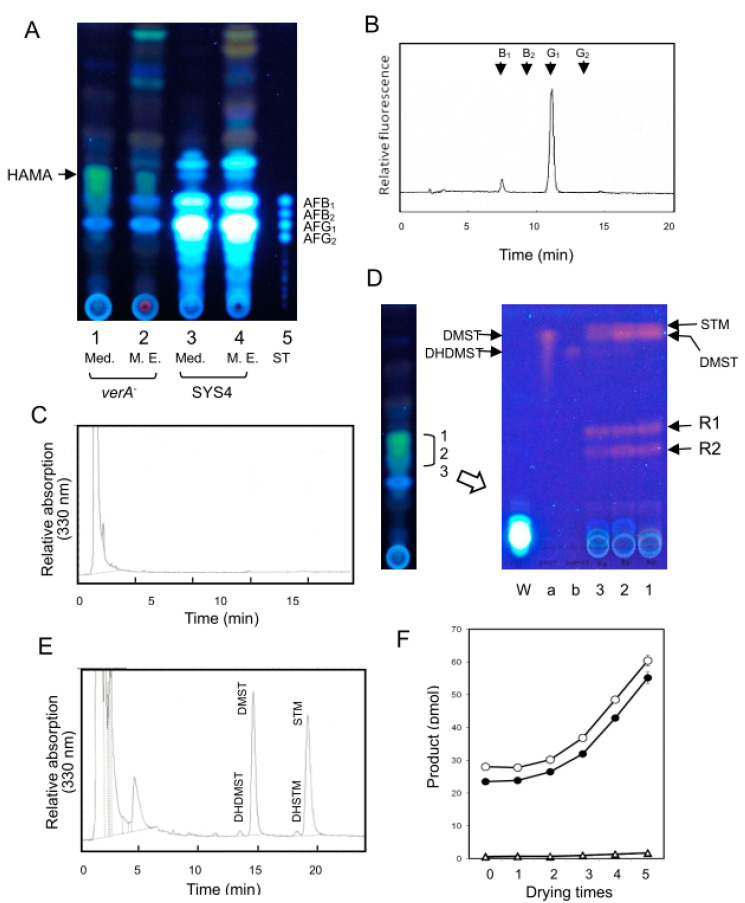
The production of a new intermediate, HAMA, and changes of the intermediate to DMST and STM. (**A**) Characterization of metabolite(s) of the *verA* disruptant. After the tip culture of the *verA* disruptant and SYS4, an extract (50 µL) of culture medium (1, 3) and mycelial extract (50 µL) (2, 4) of the *verA* disruptant (1, 2) and SYS4 (3, 4) were analyzed by TLC using solvent (**A**). (**B**) The production of AFB_1_ and AFG_1_ from HAMA by the tip culture method. NIAH-26 mutant was fed with TLC-purified HAMA pigment in YES medium, and the resulting culture medium was analyzed by HPLC using solution (**A**). (**C**) HPLC analysis of HAMA. Extract of the culture medium of the *verA* disruptant was analyzed by ODS HPLC using solution (**D**). (**D**) The change of TLC-purified HAMA to other substances. The broad yellow fluorescent band (HAMA) on the TLC plate (as shown in panel A) was divided into three parts (1–3), and each part was extracted and then re-analyzed by TLC using solution B. Four red substances (STM, DMST, R1, and R2) were observed. a, DMST; b, DHDMST. (**E**) The non-enzymatic conversion of HAMA to DMST and STM and contaminated DHHAMA to DHDMST and DHSTM. The drying of TLC-purified HAMA under N_2_ gas followed by solubilization was repeated five times, followed by an HPLC analysis as described in panel C. Standard samples of DMST, STM, and DHDMST were also analyzed. (**F**) The production of DMST and STM from HAMA by the repetition of drying and solubilization. The same procedures as those described in panel E were done from zero to five times, followed by an HPLC analysis. The amounts of DMST (closed circles), STM (open circles), DHDMST (closed triangles), and the suspected DHSTM (open triangle) were measured.

The *verA* disruptant and wildtype SYS4 were cultured by the tip culture method. The TLC analysis (Figure 3) of the culture medium showed that the *verA* disruptant accumulated a novel yellow fluorescent substance in both the culture medium and mycelia (Figure 3A). Interestingly, we observed that the yellow color of the substance on the TLC plate changed gradually to a dark red color after the plate was left overnight, suggesting that this substance may be an unstable one. The TLC analysis of the culture medium as well as that of the mycelial extract also showed that the *verA* disruptant still produced small amounts of AFs (Figure 3A), and when the extract of the culture medium was analyzed by HPLC, a negligible amount of AFB_1_ and a small amount of AFG_1_ were detected (Table 2).

To determine the step(s) involving the *verA* gene in AF biosynthesis, we performed feeding experiments using several precursors of AFs. When the *verA* disruptant was incubated with DMST, ST, or OMST, these substances were converted to AFB_1_ and AFG_1_, whereas when either VHA or VA was used, AFs were not produced (Table 3), indicating that *verA* gene is involved in a certain step between VA and DMST in the biosynthesis of AF.

For the characterization of the yellow pigment, we isolated the pigment from the TLC plates after the culture media were analyzed by TLC using solution A. When strain NIAH-26 was incubated with the pigment, significant amounts of AFB_1_ and AFG_1_ were produced (Figure 3B), indicating that the pigment is a precursor of these AFs.

We further analyzed the yellow pigment by TLC. Since the pigment spot appeared to be broad in the TLC analysis (Figure 3A), we first divided the spot into three parts (Figure 3D, Left panel), and the pigment was extracted from each part. Each extract was then analyzed by TLC using another solvent. Four dark red substances were newly detected, and the Rf values of the upper two bands were the same as DMST and STM, suggesting that the yellow pigment could be a precursor of DMST as well as STM. We herein named this pigment “HAMA” after Professor. Takashi Hamasaki. Two other substances (indicated by R1 and R2) were also detected (Figure 3D, Right panel). However, we observed that significant amounts of AFs were not produced by either R1 or R2 in feeding experiments using strain NIAH-26. We speculate that these are dead-end products produced by HAMA, and they remain to be studied.

To confirm the productions of DMST and STM from the HAMA, we used HPLC to analyze the samples directly extracted from the culture media of the *verA* disruptant (Figure 3C). A peak around 2 min was detected, whereas peaks of DMST and STM were not detected. In contrast, the TLC-purified samples (after the TLC and the scraping off of the HAMA spot, then extraction followed by drying and solubilization with a small amount of methanol) showed productions of DMST and STM (Figure 3F, 0-time drying). When the same substances underwent repeated drying with N_2_ gas followed by solubilization with methanol, the amounts of DMST and STM similarly increased with the number of repetitions (Figure 3F). The HPLC analysis of the five-repetitions samples showed that large amounts of DMST and STM were produced. Interestingly, a small amount of DHDMST and another substance were also detected, and the latter substance was suggested to be dihydrosterigmatin (DHSTM) based on the similarity of the elution patterns of DHDMST and DMST (Figure 3E). These results suggest that the HAMA sample extracted from the TLC plate were contaminated with a small amount of dihydroHAMA (DHHAMA).

We also investigated the heat stability of the HAMA pigment. After culture medium of the *verA* disruptant was autoclaved at 121 °C for 15 min, the resulting solution was fed to strain [*pks-fas-1*]^−^. When the treated medium was used, the amounts of AFs were decreased to approx. 10% of those obtained when we used the intact medium (Table 4, Exp. 1). When the same medium was treated using either acid condition (pH 1.5) or alkaline condition (pH 11.0), the amounts of AFB_1_ and AFG_1_ decreased to 50%–70% of those when we used the intact *verA* culture medium (Table 4, Exp. 2). These results suggested that HAMA is unstable against heat treatment and partially sensitive to either acid or alkaline treatment. Although small amounts of AFB_2_ and AFG_2_ were also produced from the HAMA pigment, it was suggested that HAMA pigment sample was contaminated with small amount of dihydroHAMA.

### 2.3. Relationships among the verA, ver-1, hypA and ordB Disruptants

**Figure 4:**
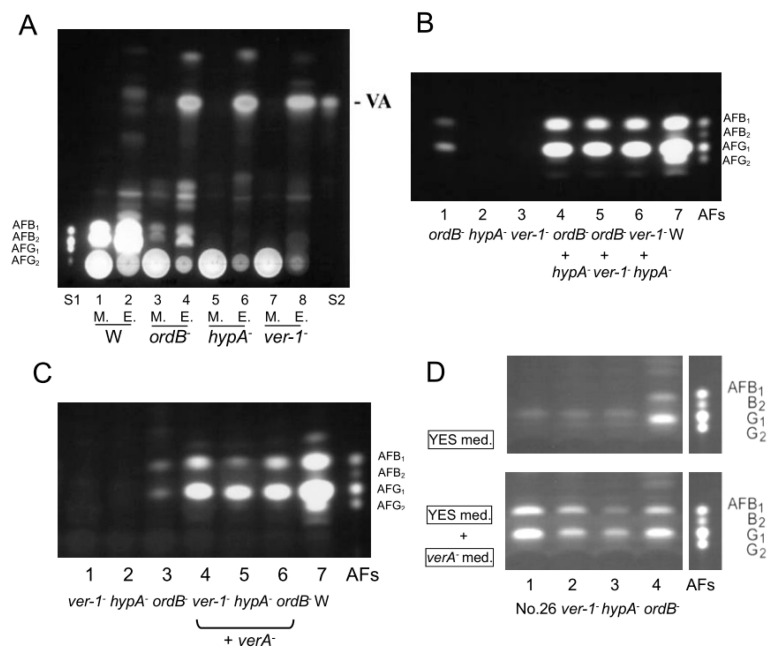
The relationships among *verA* gene, *ver*-*1*, *hypA* and *ordB* genes. (**A**) After tip cultures, culture media (1, 3, 5, 7) and mycelial extract (2, 4, 6, 8) of SYS4 (1, 2), or the *ordB* (3, 4), *hypA* (5, 6), or *ver-1* disruptant (7, 8) were analyzed by TLC using solution B. S1: AF mixture standard, S2: VA authentic sample. (**B**) TLC analysis of the AF production by each of the *ordB*, *hypA* and *ver-1* disruptants or their co-culture. The culture media (30 µL) of each of the *ordB* (1), *hypA* (2) and *ver-1* (3) disruptants, or the co-culture of *ordB* and *hypA* disruptants (4), the co-culture of *ordB* and *ver-1* disruptants (5), the co-culture of the *ver-1* and *hypA* disruptants (6) were analyzed by TLC using solution (**A**). (**C**) TLC analysis of AF production in coculture of *verA* disruptant and each of *ordB* deleted mutant (lower). The culture media of each of *ver*-1- (1), *hypA* (2) or *ordB* (3) disruptant, or the co-culture of *verA* disruptant and each of *ver*-1 (4), *hypA* (5) or *ordB* (6) disruptants and SYS4 strain (7) together with AF standard sample (AFs) were also analyzed. (**D**) The feeding of disruptants with HAMA in the culture medium of *verA* disruptant. Each of NIAH-26 (1), ver-*1* (2), *hypA* (3), and *ordB* (4) disruptants was cultured in YES medium (upper panel) or YES medium supplemented with the same volume of the culture medium of *verA* disruptant. The resulting culture medium was then analyzed by TLC.

Culture media as well as mycelial extracts of the *ver-1*, *hypA*, and *ordB* disruptants were analyzed by TLC after tip culture. They predominantly accumulated VA as a major intermediate in their mycelia (Figure 4A). Each of the disruptants also produced smaller amounts of some substances, which appeared to be different depending on the disruptant. When each substance was extracted from a TLC spot and then analyzed by HPLC, we observed that the *hypA* disruptant accumulated a small amount of 6-deoxyversicolorin A (deoxyVA) together with VA. The *ver-1* and *hypA* disruptants completely lost AF productivity, whereas the *ordB* disruptant produced small amounts of AFs (Figure 4A). To investigate relationship among *ver-1*, *hypA*, *ordB,* and *verA* genes in AF biosynthesis, we co-cultured each combination of two of them and analyzed AFs contained in the resulting culture medium (Figure 4B,C). All combination enhanced AF production, indicating that all genes have different functions each other although all accumulated mainly VA.

Since the culture medium of the *verA* disruptant contained a significant amount of HAMA pigment (Figure 3A), we investigated the AF levels in the *ver-1*, *hypA* and *ordB* disruptants fed with filter-sterilized culture medium of *verA* disruptant (Figure 4D). The addition of the medium of the *verA* disruptant significantly enhanced the productions of AFB_1_ and AFG_1_, indicating that the HAMA substance is a precursor at a certain step after any steps involving the *ver-1*, *hypA* and *ordB* genes. The productions of AFB_1_ and AFG_1_ by strain NIAH-26 were also confirmed (Figure 4D).

### 2.4. The LC-MS Analysis of HAMA Pigment

**Figure 5:**
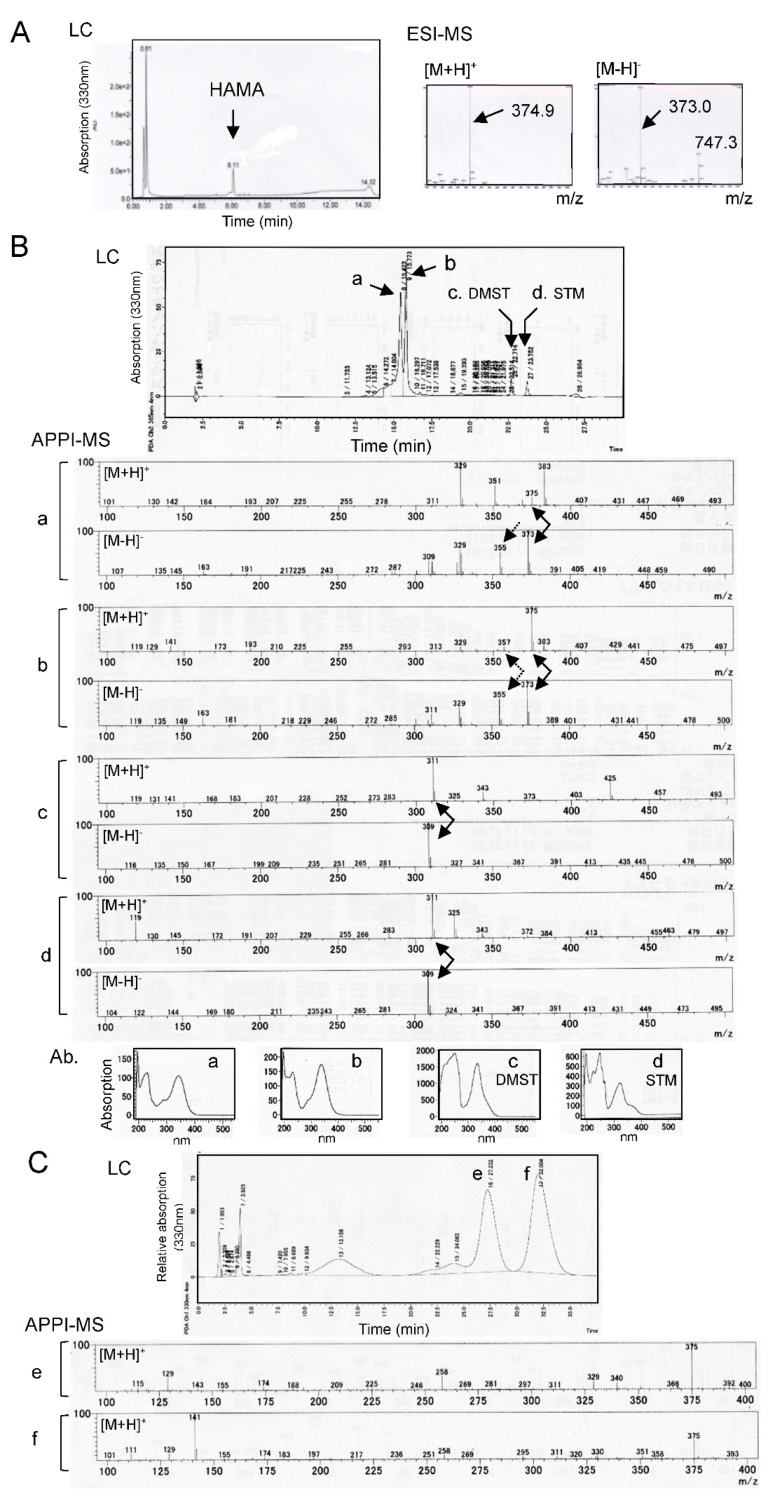
The LC-MS analysis of HAMA produced by the *verA* disruptant. (**A**) The LC-ESI-MS of HAMA pigment. After HAMA pigment was purified by Diaion HP20, the resulting substance was analyzed by LC-ESI-MS using condition [A]. HAMA (arrow) showed a single peak on LC chromatograms, and its molecular mass was indicated to be 374 on MS chromatogram. (**B**) The LC-APPI-MS of HAMA and its derivatives. The HAMA preparation after the repetition of five-times drying and solubilization (which was the same as the sample in Figure 3E,F), was analyzed by LC-APPI-MS using condition [B]. The mass spectra (middle) and absorption spectra (lower) of each peak (a~d) on the LC chromatogram (upper) are shown. The quasi-molecular ion peaks of each substance and the ion peaks caused by dehydration of the quasi-molecular ion peaks are indicated as solid arrows and dotted ones in the mass spectra, respectively. (**C**) LC-APPI-MS of the extract of the culture medium of the *verA* disruptant. After the culture medium of the *verA* disruptant was extracted with ethyl acetate, the resulting extract was analyzed by LC-APPI-MS using condition [C]. The mass spectra of peaks (e,f) on the LC chromatogram (upper panel) are shown respectively.

The LC-MS analysis using condition [A] revealed that HAMA purified by TLC had a peak whose retention time was at 6.11 min in the LC chromatogram (Figure 5A). HAMA’s molecular related ions in electrospray ionization-mass spectrometry (ESI-MS) spectra were observed at m/z 374.9 [M + H]^+^ (100%) or 373.0 [M−H]^−^ (100%) and 747.3 [2M−H]^−^ (32%), indicating that the molecular mass of HAMA is 374 (Figure 5A).

When condition [B] was used for the LC-MS measurement, HAMA after five-times drying under N_2_ showed several peaks (Figure 3E,F). Two main peaks were observed at around 15.4 min and 15.8 min on LC chromatograms, and the former peak afforded ion peaks at m/z 375 [M + H]^+^ (23%) (Figure 5B-a, Upper) and 373.0 [M−H]^−^ (100%), and 355 [M−H_2_O−H]^−^ (55%) (Figure 5B-a, lower) in atmospheric pressure photoionization-mass spectrometry (APPI-MS) spectra. The latter peak gave ion peaks at m/z 375 [M + H]^+^ (100%), 357 [M − H_2_O + H]^+^ (11%) (Figure 5B-b, upper) and 373.0 [M − H]^−^ (100%), 355 [M−H_2_O−H]^−^ (58%) in APPI-MS spectra (Figure 5B-b, Lower). These results indicated that the HAMA molecular mass is 374, and HAMA gave a dehydrated fragment of 356 during ionization. The absorption spectra of the former substance and the latter one by photodiode array (PDA) detection also demonstrated that their chromophores are similar to each other (Figure 5B, Bottom).

The retention times of other smaller peaks (22.7 min and 23.8 min) on LC chromatograms were coincident to those of authentic samples of DMST and STM, respectively, and their molecular masses were commonly 310 in APPI-MS spectra (Figure 5B-c,d). These LC-MS data demonstrated that the substances produced through the repetition of drying followed by solubilization were confirmed to be DMST and STM. We also analyzed the extract of the *verA* disruptant culture medium to examine the fresh sample of HAMA in more detail using condition [B]. We obtained the same results as those of the repeated dried sample (Figure 5B, LC) except that the peaks of DMST and STM were remarkably small.

To confirm the reproducibility of HAMA’s twin peaks, we further analyzed the same fresh extract of the *verA* disruptant culture medium by LC-MS using condition [C]. Two main peaks were observed at 27.2 min and 32.0 min on LC chromatograms (Figure 5C). The APPI-MS spectra obtained for either peak commonly gave ions of m/z 375 [M + H]^+^ (100%) (Figure 5C-e,f). These results indicated that HAMA is composed of two isomers or conformers, and their molecular masses are both 374.

### 2.5. Accumulation of Small Amounts of STM Together with DMST in A. nidulans stcP-Disruptant

**Figure 6:**
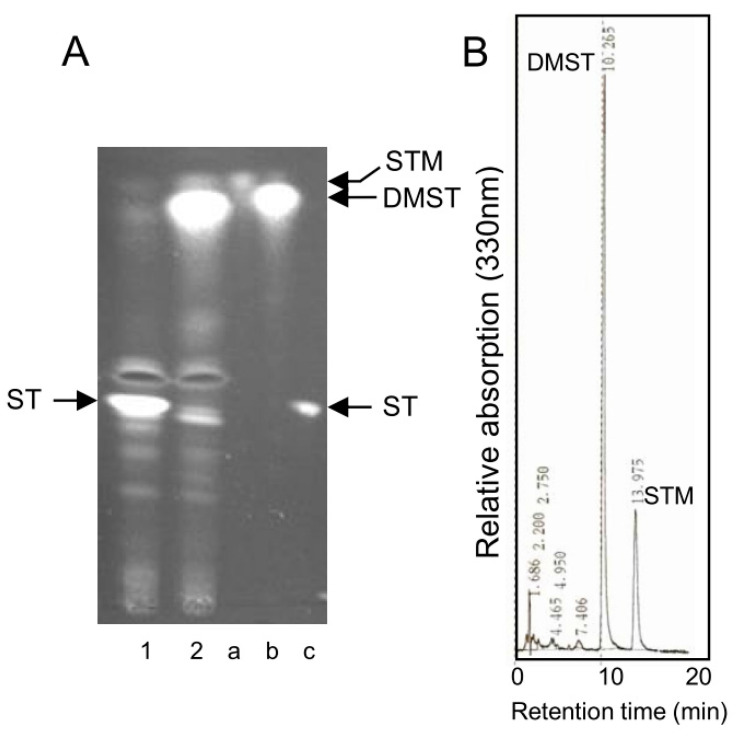
The accumulation of STM together with DMST in *A. nidulans stcP* disruptant. *stcP* gene is a homolog of *A. parasiticus dmtA.* (**A**) After *A. nidulans* FGSC26 (wild strain) (1) and TAHK64.42 *stcP* disruptant (2) were cultured in oat flakes medium for 5 days, and the resulting media were extracted with a solution containing acetone:chloroform (1:1, *v/v*). The resulting extracts were analyzed by TLC using a solution of benzene:ethyl acetate (7:3, *v/v*). The TLC plate was then sprayed with 10% aluminum chloride in ethanol followed by heating at 80 °C for 5 min. Authentic standards of STM (a), DMST (b), and ST (c) were also analyzed. Similar results were reported by Kelkar et al. (1996). (**B**) The part corresponding to the spot of STM on the TLC plate (A) was extracted and then the extract was analyzed by HPLC.

Strain TAHK64.42 is a *stcP* disruptant of *A. nidulans* FGSC26 [37], and *stcP* gene is a homolog of *dmtA* gene of *A. parasiticus* [38]. After these strains were incubated in oat flake medium, metabolites produced by either strain were analyzed by TLC (Figure 6A). Strain TAHK64.42 newly produced a large amount of DMST and a small amount of STM. The production of STM in this strain was also confirmed by an HPLC analysis (Figure 6B). The detection of STM suggested that HAMA accumulated together with DMST in the *stcP* disruptant and would be non-enzymatically changed to STM during the extraction procedure in this work. We speculate that HAMA was formed before a formation step of DMST in the biosynthesis of ST.

## 3. Discussion

### 3.1. verA Gene and HAMA Intermediate

**Figure 7:**
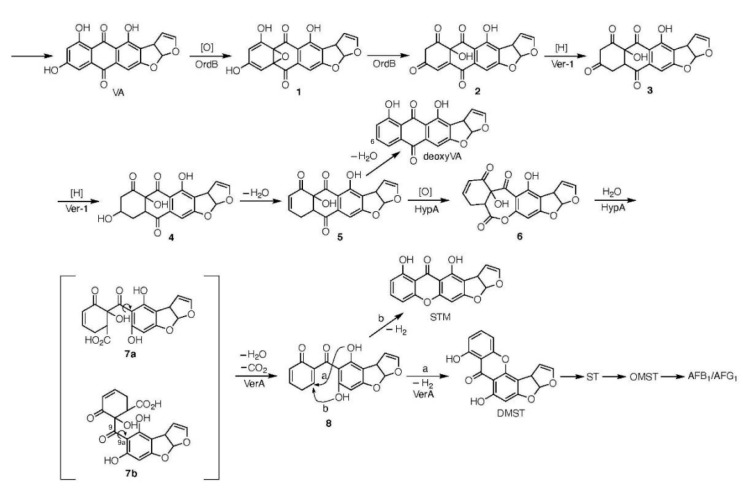
The postulated pathway of the conversion of VA to DMST via the formation of HAMA (intermediate **7**). *ver*-1, *hypA* and *ordB* are involved in the pathway from VA to HAMA, because each gene disruptant converted HAMA to AFB_1_ and AFG_1_. HAMA is shown as intermediate **7**a or **7**b based on the molecular mass (374); **7a** and **7b** are different conformers of HAMA. The LC-MS analysis showed that HAMA is composed of two types of conformers, and each conformer corresponds to either an STM-like structure and a DMST-like structure. Although either conformer of HAMA is speculated to be non-enzymatically converted to DMST or STM, VerA giostereospecifically converts either conformer to intermediate **8** through dehydration and decarboxylation and then converts the resulting intermediate **8** to DMST through dihydroxylation (pathway a). Intermediate **8** is also non-enzymatically converted to DMST (pathway a) and STM (pathway b) at the ratio of 1:1 in their quantities. The same reaction steps may be involved in the formation of DHDMST from VB except that all intermediates are dihydro-derivatives of the intermediates of the 1-group AFs.

This work demonstrated that *verA* gene is involved in building the xanthone moiety of DMST or DHDMST in the biosynthesis of AF. The postulated pathway of the conversion of VA to DMST is shown in Figure 7. The *verA* disruptant accumulated a novel intermediate, HAMA, which is a precursor of AFB_1_/AFG_1_ (Figure 3B). The molecular mass of HAMA was 374, and HAMA changed non-enzymatically to DMST and STM at the ratio of 1:1 (Figure 3E,F). Since TLC-purified HAMA also contained much smaller amounts of DHDMST and a metabolite presumed to be DHSTM (Figure 3E), DHHAMA seemed to be contaminated in the HAMA sample. We thus suspect that *verA* gene is involved in the reaction from HAMA to DMST as well as the reaction from DHHAMA to DHDMST in AF biosynthesis. STM and DHSTM have not been isolated from aflatoxigenic fungi because the HAMA and DHHAMA that are transiently formed as intermediates of AFs are immediately changed to DMST and DHDMST, and to AFs. In contrast, STM was isolated from *A. nidulans stcP* disruptant because the conversion step from DMST to ST was completely blocked by the gene disruption (Figure 6A). Kelker et al. [37] showed that a significant amount of the substance thought to be STM together with DMST was produced in the *A. nidulans stcP* disruptant. Like *A. nidulans*, *A. versicolor* species produce ST as an end-product, and Hamasaki et al. first isolated STM from *A. versicolor* (Vuillemin) Tiraboschi. The accumulations of ST and other later intermediates in ST biosynthesis may be a key to the successful isolation of STM.

*Aspergillus ustus* produces austocystins A–I as its secondary metabolites. Like STM, these substances have a linear fusion structure of the xanthone and dihydrobisfuran moieties. Since *A. ustus* does not produce DMST, another enzyme for regiospecific ring closure to make the linear structure of austocystins A–I would work in this species. A comparison of this possible enzyme to VerA enzyme would be useful for gaining a better understanding of the stereospecific reactions of the intermediates.

We also observed that the heat treatment of the culture medium of *verA* disruptant with autoclaving greatly decreased (7–13%) the production of AF when the treated culture medium was used in the feeding experiment (Table 4, Exp. 1), suggesting that heat treatment caused a disruption of HAMA’s structure. In contrast, the acid or alkaline treatment of the culture medium caused a partial decrease (89–43%) of AF production in the feeding experiments (Table 4, Exp. 2). We suspect that this partial inhibition is due to the productions of DMST and STM from HAMA by the pH changes. The structural changes of HAMA caused by these treatments remain to be studied.

The HPLC analysis revealed that the *verA* disruptant produced a slight amount of AFG_1_ (Table 2). However, the TLC analysis of the culture medium and the mycelial extract of the *verA* disruptant indicated that they produced small but significant amounts of AFB_1_ and AFG_1_, suggesting that the disruptant might be a leaky mutant (Figure 3A, lanes 1 and 2). However, these results are not easily understood because *verA* gene encodes a cytochrome P450 monooxygenase, and the reaction for making the xanthone structure seems to be too unique for another enzyme to be substituted for VerA enzyme. It is possible that a small amount of HAMA accumulates in cells of the *verA* disruptant and then non-enzymatically change to DMST in the cells, and the resulting DMST is fed by the *verA* disruptant to produce AFB_1_ and AFG_1_. A future study should determine whether the *verA* disruptant is leaky.

### 3.2. The Postulated Pathway from VA to HAMA

*A. parasiticus verA* (*aflN*) gene was originally suspected to be a homolog of *A. nidulans stcS* gene based on the homology (75%) of their deduced amino acid sequences [16]. Our present findings confirmed that the *verA* gene is not a homolog of *A. nidulans stcS* gene. The pathway from VA to DMST contains a step for the formation of the xanthone structure from the anthraquinone moiety in the biosynthesis of AF. We propose that the *ver-1*, *ordB*, and *hypA* disruptants are involved in the conversion of VA to HAMA pigment, and that the *verA* gene is involved in the reaction from HAMA to DMST (Figure 7).

Although the *ver-1* and *hypA* disruptants did not produce any AFs, the *ordB* disruptant produced small amounts of AFs (Figure 4A), which was previously reported [27]. The co-culture of two of *verA*, *ver-1*, *ordB* and *hypA* disruptants remarkably enhanced the production of AFs (Figure 4B,C), indicating that these genes have differing functions in AF biosynthesis. Ehrlich et al. [27] suggested that the common accumulation of VA by the *ver-1*, *ordB* and *hypA* disruptants may be due to their work as an enzyme complex in AF biosynthesis. In the present study, we searched for any related enzymatic activities in the pathway from VA to DMST by using cell-free systems of each disruptant, and we did not detect any significant enzyme activities. The detailed enzyme reactions remain to be investigated.

The pathway from VA to DMST has been speculated to be composed of the epoxidation of the anthraquinone moiety of VA, reduction, Baeyer-Villiger oxidation, and decarboxylation [40]. We herein hypothesize the existence of a reaction scheme in which intermediates **1–8** are involved in the pathway from VA to DMST (Figure 7). Since *ordB* is thought to encode a metal-oxidase [28], we suggest that OrdB enzyme may catalyze the epoxidation of VA and the subsequent epoxide ring-opening step of intermediate **1**. In contrast, *ver-1*gene had been suggested to encode an NADPH-dependent reductase [29]. Since the reactions from intermediates **2** to **4** are composed of two successive reduction reactions, we suspect that the Ver-1 enzyme catalyzes these reactions.

We observed that these disruptants also accumulated minor products together with VA and that the minor products were different depending on each disruptant. Among them, we determined that *hypA* disruptant accumulated a small amount of 6-deoxyversicolorin A, and the same result was obtained by Erlich et al. [27]. In contrast, *ver-1* and *ordB* disruptant accumulated small amounts of other substances, which were not identified herein. Although HypA enzyme has no typical homologous domain to other proteins, Erlich et al. [27] expected that the amino acid sequence of HypA enzyme would have partial similarity to those of some enzymes related to a Baeyer-Villiger reaction. We speculated that HypA enzyme may catalyze the reaction from intermediate **5** to intermediate **6** and the next hydrolytic ring opening of intermediate **6** to afford HAMA **7**. The involvement of HypA enzyme in this step may explain why the *hypA* disruptant produced a small amount of 6-deoxyVA together with the major product, VA. The 6-deoxyVA is suspected to have been produced from intermediate **5** through a dehydration reaction. Since strain NIAH-26 fed with 6-deoxyVA did not produce any AFs, we speculate that 6-deoxyVA is a dead-end product derived from an intermediate for AF production.

### 3.3. The Postulated Pathway from HAMA to DMST

VerA enzyme catalyzes the reactions from HAMA **7** to DMST (Figure 7). The enzyme catalyzes two steps: the concerted decarboxylation and dehydration of HAMA **7** to afford intermediate **8**, and the regiospecific ring-forming dehydrogenation of **8** to give DMST. In the LC-MS analyses, HAMA gave two peaks (Figure 5), suggesting some possibilities regarding its structural features; one is that HAMA gives two conformers (**7a** and **7b**) because of a restricted rotation around the bond with steric hindrance as well as a hydrogen bond between the ketone carbonyl and phenolic hydroxyl as shown in Figure 7. The other possibility is that HAMA has two chiral centers on the cyclohexenone moiety and gives two diastereomers. The reaction steps from VA to HAMA are enzyme-catalyzed steps and are suspected to be stereospecific. Therefore, the former has higher possibility.

Both conformers **7a** and **7b** could be changed to the same intermediate **8**. We also speculate that one of the two conformers **7a** and **7b** would be selectively bound on the substrate binding site of VerA enzyme, and then converted to intermediate **8** and DMST. The remaining conformer would be changed to the other conformer that is suitable for the VerA enzyme reaction through a rotation around the bond at C-9 and C-9a of the HAMA molecule.

Our present results demonstrated that HAMA can non-enzymatically change to DMST and STM at the ratio of ~1:1 in their quantities (Figure 3D–F and Figure 5B). The yellow color of the spots of HAMA on the TLC plates changed to dark red, which is the same color as those of ST derivatives such as DMST and STM under UV light. The repetitions of the drying and solubilization of the HAMA increased the amounts of DMST and STM. These results indicated that decarboxylation, dehydration, and dehydrogenation can occur spontaneously. Since STM did not serve as a precursor of AFs in the feeding experiment with NIAH-26 mutant, we speculate that STM is an artificial dead-end product from intermediate **8**.

Therefore, VerA enzyme is the key enzyme for the formation of the xanthone structure of various ST derivatives. Since acute toxicity and carcinogenicities have been reported after xanthone structure formation, VerA enzyme is also involved in the appearance of these toxicities. We also speculate that because aflatoxigenic fungi produce 2-group AFs (AFB_2_ and AFG_2_) together with 1-group AFs (AFB_1_ and AFG_1_), VerA enzyme is involved in the reaction from VB to DHDMST through the formation of DHHAMA.

## 4. Materials and Methods

### 4.1. Fungal Strains

*A. parasiticus* SYS4 (NRRL-2999), the wild-type AF-producing strain, was used as a recipient strain for gene disruption. *A. parasiticus* NIAH-26, a UV-irradiated mutant from strain SYS4, produces all enzymes in the AF biosynthesis pathway from norsolorinic acid to AFs, although it produces neither AFs nor any precursors [41,42]. We used the NIAH-26 mutant for the feeding experiments. We isolated disruptants of *verA*, *ver-1*, *hypA* and *ordB* genes herein and used them for the characterization of function of *verA* gene. *A. parasiticus* [*pks-fas-1*]^−^, which has the deletion of the 18-kb region from the *pks* gene to *fas-1* in the AF gene cluster of strain NR-1 [21], was used for the examination of the stability of the HAMA substance. Strains *A. nidulans* FGSC26 and TAHK64.42 (*stcP* deletion mutant of FGSC26) were a kind gift from Dr. Nancy Keller [37].

### 4.2. Metabolites

Aflatoxin B and G mixture was purchased from Sigma-Aldrich Co. (St. Louis, MO, USA). NA [43], AVN [41], and VHA were obtained as reported [44]. VA [45,46], ST [46], DMST, DHDMST [47], and STM [33] were prepared from mycelia of *A. versicolor* (Vuillemin) Tiraboschi as described in those studies. OMST was prepared by the methylation of ST with methyl iodide and sodium carbonate in acetone [48].

The concentrations of the metabolites in methanol were determined from UV absorption spectra by using the following molar absorption coefficients (*λ_max_*) [22,36]: AVN, 6700 M^−1^ cm^−1^ (453 nm); VHA, 7300 M^−1^ cm^−1^ (480 nm); VA, 8166 M^−1^ cm^−1^ (452 nm); ST, 16,900 M^−1^ cm^−1^ (324 nm); DMST, 19,100 M^−1^ cm^−1^ (335 nm); DHDMST, 19,400 M^−1^ cm^−1^ (335 nm); OMST, 16,500 M^−1^ cm^−1^ (310 nm); and STM, 16,900 M^−1^ cm^−1^ (324 nm).

### 4.3. Media and Culture Conditions

For the production of AFs and metabolites produced by fungi, YES medium (2% yeast extract, 20% sucrose) or GY agar medium (2% glucose, 0.5% yeast extract, and 2.0% agar) was used. For the culture of *A. nidulans* strains, oat flake medium (3 g of oat flakes and 0.5 mL of water) (Keller medium) was used.

For the standard culture followed by a thin-layer chromatography (TLC) or high-performance liquid chromatography (HPLC) analysis, the tip culture method was used [41]. Spores (approx. 1 × 10^−4^) of each SYS4 and various gene disruptants were inoculated into 250 µL of YES medium in a 1-the ml pipetman tip. After culture at 28 °C for 4 days, culture media and the mycelia were separated by centrifugation and then used for further analyses. For the preparation of larger amounts of intermediates for the physico-chemical analyses of HAMA, the *verA* disruptant was cultured in 100 mL of YES medium at 28 °C for 4 days [49]. Aliquots of the medium were used for the TLC and the LC-MS.

### 4.4. Preparation of verA Disruptant

To construct the *verA* disruption vector, three steps were taken: the 2-kb *Pst*I/*Kpn*I polymerase chain reaction (PCR) fragment (pcr1) of the selectable marker gene *ptrA* [50] was amplified with primer pair P19/P20 (Table 1) and inserted into the corresponding sites in the pSP72 vector (Figure 2B). Then, a 1.2-kb fragment of the upstream of *verA* and the 3′ coding region of *ver-1* (pcr2) and a 1.45-kb fragment of the downstream of *verA* and the 3′ coding region of *avnA* (pcr3) were amplified using *A. parasiticus* SYS4 genomic DNA as the template. The two primer pairs P7/P8 and P9/P10 with their restriction endonuclease sites are underlined and the enzymes are indicated in parentheses in Table 1. The *Xho*I-*Pst*I fragment from pcr2 and the *Kpn*I-*Bgl*II fragment from pcr3 were cloned into the corresponding sites in the pSP72 bearing *ptrA* to give the disruption vector, pVERA-DD. The sequence of pVERA-DD was checked by a restriction analysis. For transformation, pVERA-DD was linearized with *Cfr*9I to release the 4.5-kb insertion part from the pSP72 vector.

For fungal transformation, the preparation of the protoplasts from *A. parasiticus* SYS4 and the transformation with DNA were performed as described by Wen et al. [49] with some modifications. For the transformation of *Cfr*9I-digested pVERA-DD, 4–8 µg of DNA was added to 0.1 mL of protoplasts. The pyrithiamine (PT)-resistant transformants were screened on CDA regeneration medium with 0.1 µg mL^−1^ pyrithiamine [50]. Approximately 90–120 PT-resistant transformants per 6 µg of DNA was obtained. The transformants were then transferred to a GY agar plate and cultured.

The desired *verA* disruptant was then selected by assessing the impairment of AF production by UV photography [51]. On a UV picture taken under 365-nm UV light, the AF production-impaired disruptants appeared as white colonies, and the AF-producers were identified as gray or black colonies. Confirmation of the deletion was performed by PCR analysis. DNA of the white mutant was prepared using FastPrep FP100A (Qbiogene, Carlsbad, CA, USA) as described [49].

To investigate the replacement of the *verA* gene with the *ptrA* marker gene by PCR, we used three PCR primer pairs (Table 1): P3/P6 and P4/P5 for testing the insertion of *ptrA* into SYS4 genomic DNA, and P1/P2 for testing the deletion of *verA.* After we compared the PCR results for transformants with those for the recipient strain, the selected *verA* disruptant was purified three times by single colony isolation on GY plates. The mutation in these selected *verA*^−^/SYS4 transformants was re-confirmed by PCR. The deletion of the *verA* gene in the genome was also confirmed by a Southern analysis.

Total genomic DNA of *verA* mutant and strain SYS4 was subjected to restriction enzyme digestion with *Eco*RI and separated by agarose gel electrophoresis, followed by blotting to a Hybond-N+ membrane (GE Healthcare, Buckinghamshire, UK). The filters were hybridized with the *verA* and *ptrA* probes. The 0.83-kb *verA* probe was amplified from genomic DNA using primer pairs verA-F442 [5′-GTTTCGACTCCCTCGGC-3′] and verA-R2922 [5′-TGCGGCCCTGAGCTTCT-3′]. The 2.0-kb *ptrA* probe was amplified from pPTRI plasmid (Takara Bio, Shiga, Japan) using the primers as described [52]. Hybridization and detection were performed using the Alkphos Direct Labelling and Detection System (GE Healthcare, Chicago, IL, USA) according to the supplier’s manuals.

### 4.5. Preparation of ver-1, ordB, and hypA Deletion Mutants

To delete the *ordB* gene in the genome of *A. parasiticus* SYS4, we constructed the *ordB* gene disruption plasmid, pORDB-L/R, in a two-step procedure. A 1.4-kb fragment (ordB-R) of the 3′-flanking region of *ordB* gene including the 3′ non-coding sequence of the gene and the coding sequence of *moxY* gene and a 1.4-kb fragment (ordB-L) of the 5′-flanking region of the *ordB* gene including the 3′-non-coding region and the coding sequence of *hypA* gene were amplified by PCR using two sets of primer pairs with endonuclease restriction sites underlined to facilitate sub-cloning. Pair 1: ordB-L-F [5′-GAAGACCGCGGAGAATGG-3′] and ordB-L-KpnI-R [5′-CGGGGTACCGCCCACCTCTTCGTACCTAG-3′]. Pair 2: ordB-R-HindIII-R [5′-CCCCAAGCTTGCAATTGTGTAGTCTTCTCTGG-3′] and ordB-R-SalI-F [5′-AACGCGTCGACGTTGACGGAGGATCTGTTAGC-3′].

The resulting PCR products were successively cut with *Kpn*I for the 3′-flanking region and *Hind*III/*Sal*I for the 5′-flanking region, and then inserted into the pSP72-ptrA*Kpn*I/*Eco*RV and *Hind*III/*Xho*I sites to give a pORDB-L/R, which was then linearized by *Bgl*II and *Eam1*105I. The final linear replacement construct, which contained the 2-kb selectable marker *ptrA* flanked by a 1.4-kb fragment of the 3′-flanking region and a 1.35-kb fragment of the 5′-flanking region was used for transforming the SYS4 strain. Fungal protoplast preparation and transformation with DNA were performed as described [49]. The PT-resistant transformants were screened for CDA regeneration medium with 0.1 mg L^−1^ PT as described above or as described by Kubodera et al. [50] and then transferred to a GY agar plate to detect decreases in AF production and accumulations of the precursors. The genomic DNAs of the resulting RT-resistant transformants were prepared for a PCR analysis to confirm the detection of the *ordB* gene in the transformants.

To delete the *hypA* gene in the genome of *A. parasiticus* SYS4, we constructed the *hypA* gene disruption plasmid, pHYPA-L/R, in a two-step procedure. A 1.3-kb fragment (hypA-R) of the 3′-flanking region of the *hypA* gene including the 3′-non-coding sequence of the gene and the coding sequence of *ordB* gene and a 1.4-kb fragment (hypA-L) of the 5′- flanking region of the *hypA* gene including the 5′-non-coding region of the gene and the coding sequence of *nadA* gene were amplified by PCR using two sets of primer pairs with endonuclease restriction sites underlined to facilitate sub-cloning. Pair 1: hypA-L-SalI-R [5′-AACGCGTCGACGGTTCTGCTTGGCTGGG-3′] and hypA-L-HindIII-F [5′-CCCCAAGCTTCATGATACAGATTGAGTGCGAC-3′]. Pair 2: hypA-R-KpnI-F [5′-CGGGGTACCCGTATCTCAGTTATGCAATGTCTC-3′] and hypA-R-R [5′-AGTCCAATGCCGTCAAC-3′].

The resulting PCR products were successively cut with *Sal*I/*Hind*III for the 3′-flanking region and *Kpn*I for the 5′-flanking region, and then inserted into the pSP72-ptrA *Xho*I/*Hind*III and *Kpn*I/*Eco*RV sites to give a pHYPA-L/R, which was then linearized by *Bgl*II. The final linear replacement construct, which contained the 2-kb selectable marker *ptrA* flanked by a fragment of the 3′-flanking region and a fragment of the 5′-flanking region was used for transforming the SYS4 strain. The transformation, screening, and characterization of the PT-resistant transformants were performed as described above.

To delete the *ver-1* gene in the genome of *A. parasiticus* SYS4, we made the *ver-1* gene disruption construct in a two-step procedure. A 1.1-kb fragment of the 5′-flanking region of *ver-1* gene including the 5′ non-coding upstream sequence of the gene and the coding sequence of *norA* gene and a 1.1-kb fragment of the 3′-flanking region of the *ver-1* gene including the 3′-non-coding downstream region of *ver-1* and the coding sequence of *verA* gene were amplified by PCR using two sets of primer pairs with endonuclease restriction sites underlined to facilitate sub-cloning. Pair 1: norA-XhoI-F [5′-ATGCATTTGCTCGAGCCAACGGACTTACCC-3′] and ver-1-up-PstI-R [5′-AAAACTGCAGCTCTGCCTCTATCCAAAGCC-3′]. Pair 2: ver-1-down-KpnI-F [5′-GGGGTACCCGCTATATACTCGTGGGTGA-3′] and verA-BglII-R [5′-GCCGGACGAGAATTGCTGGGAGATCTTCAG-3′]. A 2-kb selectable marker *ptrA* gene was amplified by PCR using the primer pairs pPTRI-KpnI-F [5′-GGGGTACCGGGCAATTGATTACGGGATCCCA-3′] and pPTRI-PstI-R [5′-AAAACTGCAGTGACGATGAGCCGCTCTTGC-3′] and pSP72-ptrA. The resulting three PCR products were combined by ligase, and the final linear replacement construct was used for transforming SYS4 strain. The transformation, screening, and characterization of the PT-resistant transformants were performed as described above.

### 4.6. TLC and HPLC Analyses of AFs

After the tip cultures of SYS4 or each of the disruptants and then centrifugation, metabolites in the culture medium were extracted with an equal volume of chloroform, and the resulting extract was analyzed by TLC or HPLC. For detection of metabolites in mycelia, the mycelia in the tip was extracted with 800 µL acetone, and the resulting acetone extract was air-dried by keeping it at room temperature in dark condition. The resulting debris was solubilized by adding 100 µL of benzene:acetonitrile (98:2, *v/v*), and the extract was analyzed by TLC.

The extracts of culture medium and mycelial extract were spotted onto a silica gel TLC plate (Silica Gel 60; Merck, Darmstadt, Germany) and then developed with solution A containing chloroform:ethyl acetate: 90% formic acid (6:3:1, *v/v/v*). Fluorescence of the metabolites on the TLC plate was observed under 365-nm UV light and photographed with an Olympus C-745 digital camera. In some cases, the TLC plate was observed under 300-nm UV light and photographed with Fluor-S™ MultiImager (Bio-Rad, Hercules, CA, USA) for the detection of AFs. For the characterization of substances derived from HAMA or metabolites, strain NIAH-26, *ordB*-, *hypA*-, and *verA*-disruptants were also analyzed by TLC using solution B containing benzene:ethyl acetate (8:2, *v/v*) with 10% acetic acid.

For the measurement of the amounts of AFs using HPLC, the chloroform extract of the medium was analyzed with an HPLC apparatus (model SCL-10Avp; Shimadzu, Kyoto, Japan) equipped with a fluorescence monitor (excitation wavelength, 365 nm; emission wavelength, 425 nm; model RF-535, Shimadzu, Kyoto, Japan) and a silica gel column (0.46 × 15 cm; Shim-pack CLC-SIL; Shimadzu, Kyoto, Japan) at 35 °C. The flow solvent was solution C: toluene, ethyl acetate, formic acid (90%), and methanol (178:15:4:3, *v/v/v/v*), and the flow rate was 1 mL min^−1^ [21]. The retention times of the AFs were compared with those of standard samples (aflatoxin B-aflatoxin G mixture; Sigma-Aldrich, St. Louis, MO, USA).

### 4.7. Feeding Experiments for Aflatoxin Production

We used the tip culture method for the feeding experiment (Figure 3A). Conidiospores (approx. 1.5 × 10^5^) of either *verA* disruptant or strain NIAH-26 was inoculated into 250 µL of YES medium supplemented with 30 µM VHA, VA, DMST, ST, or OMST and cultured at 28 °C for 4 days. The AFs in the media were measured by HPLC. For the feeding experiment with HAMA, strain NIAH-26 was incubated by tip culture using YES medium supplemented with TLC-purified HAMA. In addition, NIAH-26, *ver-1* disruptant, *hypA* disruptant, and *ordB* disruptant were each individually incubated in tip cultures using YES medium supplemented with an equal volume of filter-sterilized 4-day cultured medium of *verA* disruptant for 4 days. The resulting medium was analyzed by either TLC or HPLC.

### 4.8. Co-Incubation of Two Disruptants among verA, ver-1, ordB, and hypA Disruptants

Conidiospores (approx. 1.5 × 10^5^) of each two of four *verA, ver-1*, *ordB* and *hypA* disruptants were inoculated into the same tip culture and incubated at 28 °C for 4 days. After the separation of culture medium from mycelia by centrifugation, the culture medium was analyzed by silica-gel TLC.

### 4.9. Preparation of HAMA Pigment

For the preparation of HAMA pigment, the culture medium of the *verA* disruptant was extracted with an equal volume of ethyl acetate, and the resulting extract was used for TLC or LC-APPI-MS analysis. For the purification of HAMA, HAMA pigment in the culture medium of the *verA* disruptant was purified by using Diaion HP20 (Mitsubishi Chemical Co. Tohyo, Japan), and the resulting extract was concentrated and used for LC-ESI-MS. For small scale purification of HAMA pigment, the extract of the culture medium of *verA* disruptant was analyzed by TLC, and the yellow part (HAMA) on the TLC plate was scraped and then extracted with ethyl acetate. The resulting extract was concentrated and then used for characterization of HAMA.

### 4.10. Characterization of HAMA Pigment

For the detection of the formations of DMST and STM from HAMA, we further analyzed HAMA pigment (which had been purified by TLC using solution A) by performing a TLC analysis using solution B again (Figure 3D). We measured the ethyl acetate extract of the culture medium of the *verA* disruptant or the TLC-purified HAMA by HPLC using the SCL-10Avp HPLC apparatus equipped with a UV-VIS spectrophotometric detector (model SPD-6AV, Shimadzu, Kyoto, Japan) and an octadecyl silane column (0.46 × 15 cm; STR ODS-II; Shinwa Chemical Industries, Kyoto, Japan) at room temperature. Absorption at 330 nm was monitored, and the solvent system was solution D: 75% methanol aqueous solution with a flow rate of 1 mL min^−1^ by monitoring absorption at 330 nm. The retention times of DHDMST, DMST, and STM were compared with those of the authentic standards (13.8 min, 15.0 min, and 19.8 min), respectively.

To test the stability of HAMA, we examined the heat stability of the pigment after the culture medium of the *verA* disruptant was autoclaved at 121 °C for 15 min and then cooled. The [*pks-fas*]^−^ strain was cultured with the treated HAMA, and the resulting culture medium was analyzed by HPLC. The acid and alkaline stabilities of the pigments were also examined by changing the pH of the culture medium of *verA* disruptant to pH 1.5 or pH 11.0 by adding 1 M HCl or 1 M NaOH, followed by incubation at room temperature for 3 h. After the pH of the solution was readjusted by adding the same volume of 1 M NaOH or 1 M HCl, respectively, the resulting solution was used for the feeding experiment with strain NIAH 26 using the tip culture method.

For the determination of the effect of the repetition of drying followed by solubilization, we dried the extract of the TLC-purified HAMA after the elution of the TLC spot with ethyl acetate with N_2_ gas in a microtube; the debris was then solubilized in methanol. These procedures of drying and solubilization were repeated several times. The resulting solutions were analyzed by HPLC using the ODS column and solution D.

### 4.11. LC-MS Measurements

The pigment HAMA that accumulated in *verA* disruptant was analyzed by liquid chromatography-mass spectrometry (LC-MS) using three conditions as follows:

Condition [A]: HPLC condition column: YMC-UltraH Pro C18 (2.0 mm I.D. × 50; YMC America, Allentown, PA, USA), solvent A: 0.1% formic acid in water, solvent B: 0.1% formic acid in CH_3_CN; gradient: 0 min 10% B, 2 min 10% B, 10 min 100% B, 13 min 100% B, 15 min 10% B; Detection: DAD 220–340 nm MaxPlot; MS + ESI (m/z 100–1000), −ESI (m/z 100–1000).

Condition [B]: The LC-MS 2010A system (Shimadzu, Kyoto, Japan) consisted of an LC-VP separation module equipped with an SPD-M10AVp photodiode array (PDA) detector and a single-quadrupole mass spectrometer with an atmospheric pressure photoionization (APPI) source probe. Solvent A, 5 mM ammonium acetate in water; solvent B, methanol; gradient: 0 min 10%, 2 min 10%, 17 min 95%, 22 min 95%, 23 min 10%, and 30 min 10%. MS + APPI (m/z 100–500), −APPI (m/z 100–500) An Inertsil column (150 mm × 2.1 mm, 5 mm, Supelco, Bellefonte, PA, USA) was used, and the flow rate was 0.2 mL min^−1^.

Condition [C]: the LC-MS conditions were the same as those in condition [B] except that the carrier solvent was set as an isocratic mixture of methanol and 5 mM ammonium acetate in water (40:60, *v/v*).

### 4.12. Substances Accumulated by the stcP Disruptant of A. nidulans

We analyzed the metabolites produced by *A. nidulans* TAHK64.42 (*stcP* disruptant) and FGSC26 (the wild strain) as described [37] with minor modifications. Conidiospores (approx. 1.5 × 10^5^) of either strain were cultured in oat flake medium (3 g oat flakes and 0.5 mL water) at 28 °C for 5 days. Metabolites of fungus ware extracted twice with the same volume of acetone-chloroform solution. The resulting pooled extract was concentrated and then analyzed by silica-gel TLC using benzene:acetic acid (95:5, *v/v*). Compounds on the TLC plate were visualized by spraying 10% (*wt./vol*) aluminum chloride solution in ethanol and heating. For the quantitative measurement of the metabolites, the part corresponding to either spot of STM or DMST was scraped and extracted with ethyl acetate. The extract was then analyzed by HPLC using an ODS column and solution D.

## 5. Conclusions

We clarified that *verA* gene in the aflatoxin gene cluster is involved in the step to make the xanthone structure of demethylsterigmatocystin as well as dihydrodemethylsterigmatocystin in aflatoxin biosynthesis.

## Figures and Tables

**Table 1:** Primers used in this report.

Primer	Sequence	Position on AY371490	DNA
P1	5′-GTTTCGACTCCCTCGGC	44119–44135	*verA*
P2	5′-CTCATCGTACGCTGGCG	45349–45333	*verA*
P3	5′-TGGGATCCCGTAATCAATTGCCC	38–16 *	*ptrA*
P4	5′-GCAAGAGCGGCTCATCGTCA	1998–2017 *	*ptrA*
P5	5′-ATGTCGGATAATCACCGTTTAG	42020–42041	*ver-1*
P6	5′-AACCCGAGCCATCTGCACCA	47140–47121	*avnA*
P7	5′-CCCCGCTCGAGTTTCACCGATGAGCAGGTAG (*Xho*I) **	42696–42715	*ver-1*
P8	5′-AAAACTGCAGGGAGAGTACCAGGTTCGCTT (*Pst*I) **	43894–43875	*verA*-up
P9	5′-GGGGTACCCCTGCTGTTACGAGCTATTC (*Kpn*I) **	45542–45561	*verA*-down
P10	5′-GAAGATCTGCAAAGTAACCGCATCGTGC (*Bgl*II) **	46994–46975	*avnA*
P11	5′-ATGGTTCTCCCTACTGCTCC	39983–40002	*norA*
P12	5′-CATTTTGAGGCAGAACCAAAG	41208–41187	*norA*
P13	5′-AGGCTCAGTCACTTGTTCC	41368–41386	*norA*-down
P14	5′-TTATCGAAAAGCGCCACCAT	42919–42900	*ver-1*
P15	5′-GGTCCGATGCTGAACGG	46718–46734	*avnA*
P16	5′-CATAGTCCCTGAGGCGG	47828–47812	*avnA*
P17	5′-GCGTAGGCCAGATTGCG	48662–48678	*verB*
P18	5′-GGTCCACTGCTATGGCG	49947–49931	*verB*
P19	5′-GGGGTACCGGGCAATTGATTACGGGATCCCA (*Kpn*I) **	16–38 *	*ptrA*
P20	5′- AAAACTGCAGTGACGATGAGCCGCTCTTGC (*Pst*I) **	2017–1998 *	*ptrA*

* Position on GenBank accession number AF217503. ** The added restriction site is underlined.

**Table 2:** Aflatoxin production by *verA* deletion mutant and its wild strain.

Strain	AFs (ng/250 µL Culture Medium) ^1^			
	AFB_1_	AFB_2_	AFG_1_	AFG_2_
*verA* disruptant	n.d.	n.d.	0.1 ± 0.0	n.d.
SYS4	363 ± 12	17 ± 1	1056 ± 71	30 ± 2

^1^ Tip culture using 250 µL YES medium at 28 °C for 4 days. AFs in culture medium were measured by HPLC. Values are means ± differences. n.d.: not detected.

**Table 3:** Feeding experiments of *verA* deletion mutant or NIAH-26 with some precursors of aflatoxins ^1^.

Strain	Total Concentrations of AFs Formed (ng/250 µL Culture Medium) ^2^				
	VHA	VA	DMST	ST	OMST
*verA* ^−^	n.d.	n.d.	13.1 ± 0.0	64.7 ± 3.2	165.4 ± 3.2
NIAH-26	18.4 ± 2.5	11.0 ± 0.2	14.0 ± 0.7	47.0 ± 0.2	169.4 ± 2.9

^1^ The final concentrations of all the precursors are 30 µM. ^2^ Values are means ± standard deviations; for VHA, total concentrations of AFB_1_, AFB_2_, AFG_1_ and AFG_2_; for other precursors, total concentrations of AFB_1_ and AFG_1_. n.d.: not detected.

**Table 4:** Feeding experiments of atoxigenic mutants with HAMA pigment treated with various conditions.

Strain	AFs (ng/250 µL Culture Medium) ^1^			
	AFB_1_	AFB_2_	AFG_1_	AFG_2_
Exp.1: [*pks-fas-1*]^−^ strain				
Intact	136 ± 3 (100%)	5 ± 0 (100%)	230 ± 2 (100%)	5 ± 0 (100%)
Heat treatment	10 ± 1 (7%)	1 ± 0 (11%)	13 ± 1 (6%)	1 ± 0 (10%)
Exp.2: NIAH-26 mutant				
Intact (DW)	210 ± 8 (100%)	4 ± 0 (100%)	129 ± 8 (100%)	2 ± 0 (100%)
Acid (pH 1.5)	185 ± 19 (89%)	4 ± 1 (96%)	82 ± 36 (64%)	2 ± 1 (74%)
Alkaline (pH 11)	102 ± 0 (49%)	2 ± 0 (50%)	55 ± 8 (43%)	1 ± 0 (46%)

^1^ Tip culture using 250 µL YES medium at 28 °C for 4 days. AFs in culture medium were measured by HPLC. Values are means ± differences.

## Abbreviations

AF	aflatoxin
HAMA	a novel precursor of AF, which accumulates in the mycelia as well as the culture medium of the *verA* disruptant
VA	versicolorin A
DMST	demethylsterigmatocystin
STM	sterigmatin
PCR	polymerase chain reaction
TLC	thin-layer chromatography
HPLC	high-performance liquid chromatography
LC-MS	liquid chromatography-mass spectrometry
DNA	deoxyribonucleic acid
